# Choir Mitigates Distress for Caregivers of Those With Dementia: The Voices in Motion Project

**DOI:** 10.1177/15333175251395437

**Published:** 2025-11-06

**Authors:** Nicholas Tamburri, Cynthia McDowell, Carren Dujela, Mariko Sakamoto, Denise S. Cloutier, Jodie R. Gawryluk, Andre P. Smith, Debra J. Sheets, Robert S. Stawski, Stuart W. S. MacDonald

**Affiliations:** 1 Department of Psychology, 8205 University of Victoria, BC, Canada; 2 Institute on Aging and Lifelong Health, 8205 University of Victoria, BC, Canada; 3 School of Nursing, 8205 University of Victoria, BC, Canada; 4 Department of Geography, 8205 University of Victoria, BC, Canada; 5 Department of Sociology, 8205 University of Victoria, BC, Canada; 6 Human Development and Family Studies, 4606 Utah State University, Logan, UT, USA

**Keywords:** dyadic music intervention, multilevel piecewise regression, caregiver distress, longitudinal, measurement burst design, dementia choir

## Abstract

Music-based interventions show promise for attenuating caregiver distress (CD) in informal dementia caregivers; however, research on comparable dyadic interventions is limited. This study aimed to provide a novel evaluation of whether a dyadic choral intervention could facilitate reductions in CD across 2 choral seasons. 30 caregiving dyads participated in a dementia choir across 2 ∼3.5-month choral seasons separated by a ∼4-month summer break: a naturalistic ABA design. Repeated assessment of the Zarit Burden Interview yielded up to 7 assessments of CD across the 2 choral seasons. Results showed that CD significantly declined across a participant’s first choral season, significantly rebounded to new highs upon returning from a summer break, and began to decline again; though, this latter trajectory was not significant. These results highlight the effectiveness of dyadic, music-based interventions for attenuating CD in dementia caregivers, and provides a novel methodological paradigm for use in future research.

Recent projections estimate that individuals aged 60 years and older will account for more than 20% of the global population by the year 2050, highlighting a significant increase in the population of older adults.^1^ Accompanying this rising demographic trajectory is an increasing prevalence of aging-related diseases, including Alzheimer’s disease and related dementias (ADRD) – the leading cause of long-term disability and dependency in individuals over 60 years of age.^2^ If modifiable risk and protective factors are not prioritized and addressed in a timely fashion, the prevalence of individuals living with ADRD worldwide is estimated to increase from the current 55 million cases to 139 million cases by the year 2050.^3^

The growing prevalence of ADRD will increase demands on both formal and informal care systems, with a disproportionate responsibility falling to unpaid caregivers who currently provide over 80% of dementia-related care to persons with ADRD.^4^ Informal caregivers, often family members to those living with ADRD, devote an average of 4-8 hours of care per day.^4^ In the United States, this amounts to roughly $340 billion worth (∼18 billion hours) of annual unpaid care, which is $40 billion more than the total annual economic costs associated with formal dementia care.^5^ This substantial, unpaid care provided by formal caregivers thus represents a cornerstone of dementia care relied on by both individuals living with ADRD and formal healthcare systems; yet, the litany of known adverse physical and mental health effects associated with dementia caregiving threatens the ability for such caregivers to maintain their role in a healthy and sustainable way.^4,6-9^ That is, unless interventions focused on addressing caregiver health are adopted and widely implemented, as cases of dementia continue to rise so too will healthcare issues regarding dementia caregivers, presenting a two-fold challenge to formal healthcare systems.

## The Impact of Informal Caregiving for Persons with ADRD

Informal caregiving is associated with a myriad of responsibilities that become progressively more complex and intensive as dementia progresses. For instance, in the early stages of the disease process, caregiving responsibilities often include managing higher order and complex life functions such as handling household finances and administering medications. However, as the care recipient’s disease progresses, caregiving responsibilities become increasingly centered around fundamental activities of daily living and self-care (eg, bathing, dressing, eating).^4,5^ Critically, as the time- and labor-demands of these responsibilities increase so too does the emotional impact of caregiving. That is, in addition to managing the worsening physiological and behavioral symptomatology, caregivers also often witness the gradual loss of autonomy and personhood in their loved 1 (most often a parent or spouse) and may struggle to cope with the inevitable progression of underlying neuropathology. This is underscored by the disproportionately high rates of major depressive symptomatology in caregivers, which can affect over 50% of informal carers.^10^ The combination of these substantial logistical and emotional challenges can therefore exert a significant impact on caregiver health and well-being, placing caregivers at increased risk of numerous subsequent health issues.

An extensive literature demonstrates strong associations between informal caregiving and physical health (eg, cardiovascular disease and mortality risk^7,9,11^), psychological well-being (eg, depression, anxiety^10,12^), as well as deficits in cognition including learning, working memory, episodic memory, vocabulary, and attention.^13-15^ Informal caregivers of persons with ADRD (especially spouses) are also found to be at an increased risk of developing dementia themselves relative to their non-caregiving peers.^16^ The mechanisms underlying these multisystem deficits may be related to the combined effects of chronic stress, social isolation, physical exertion, and poor sleep that are often associated with caregiving. The biological correlates of such behaviors include elevated stress hormones and inflammatory markers, hypertension, and metabolic syndrome – all of which may independently contribute to physiological and mental health deficits – and all of which are often increased in dementia caregivers relative to non-caregiving controls.^17^

Caregiver stress in particular represents a robust predictor of adverse health outcomes, as high levels of stress – associated with chronically elevated cortisol levels – contributes to a deluge of deleterious health outcomes including cognitive impairment, disturbed sleep, obesity, hyperinsulinemia, inflammation, depressive symptoms and signs, mortality risk, and neurodegeneration associated with ADRD pathology.^17-19^ As such, interventions aimed at mitigating caregiver stress may be critically important for preserving caregiver health and well-being.

## Caregiver Distress and Dyadic Health Implications

Caregiver distress (CD) is an important operationalization of caregiver stress, focusing on the caregiver’s appraisal of burden. Specifically, CD refers to the combination of both the objective and subjective experiences of caregiving; subjective distress pertains to the caregiver’s own evaluation of the stress and anxiety associated with their situation whereas objective distress refers to the physical disruption of a caregiver’s routines, habits, and household as a result of providing care.^4^ Caregivers can experience significant distress due to the combination of chronic psychological and physical strain, prolonged exposure to high stress, disruptions to sleep and daily routines, restricted leisure time and self-care, as well as the social isolation and loneliness that often accompany caring for a loved 1 with ADRD.^20-27^ Spousal caregivers in particular may exhibit higher CD relative to other caregivers,^28^ as they (i) typically provide up to 4 times the amount of care compared to non-spousal caregivers,^29^ (ii) are more likely to have age-related disability or disease,^30^ and (iii) may lack opportunities for social engagement.^25,31^ Moreover, several previous studies have demonstrated CD as a mediating variable between caregiving and episodic working memory deficits,^14^ psychological distress,^32^ and mortality risk,^7^ suggesting that modifying CD may promote a series of notable downstream health benefits to caregivers.

Critically, CD not only impacts the well-being of caregivers but care recipients as well, with elevated CD potentially compromising the level of care able to be provided and adversely affecting the care recipient’s disease trajectory. A recent systematic review reported that increasing CD is often associated with worsening mood, cognition, quality of life, and behavioral and psychological symptoms of dementia within care recipients.^33^ Additional research highlights how CD is also associated with accelerated rates of institutionalization and increased experiences of elder abuse.^34^ The serious implications of CD on the health and well-being of both the caregiver and their care recipient thus highlights the critical importance of identifying effective interventions that can modulate CD within caregivers.

## Non-Pharmacological, Music-Based Interventions

Non-pharmacological interventions are increasingly being adopted as first-line treatment strategies to help improve the health and longevity of both persons with ADRD and their informal caregivers, with social prescription being an important aspect of holistic dementia care. Indeed, recent reviews and meta-analyses affirm the utility of non-pharmacological interventions for protecting against social isolation and loneliness,^35^ attenuating the presence of behavioral and psychological symptoms of dementia in persons with ADRD and their impact on caregivers,^36^ and mitigating caregiver distress,^37^ while also bolstering cognitive functioning in individuals with ADRD.^38^ Barring a cure or effective pharmaceutical interventions that halt ADRD progression, non-pharmacological lifestyle interventions represent a critical avenue of care, addressing several salient challenges and adverse symptoms faced by both individuals with ADRD and their caregivers.

Among the different non-pharmacological interventions aimed at alleviating CD (eg, psychoeducation, physical exercise, cognitive behavioral therapy, support groups, etc.), music-based interventions have garnered significant popularity over the past decade, and their promise has been emphasized in recent policy reports from the World Health Organization.^39^ Music interventions offer increased accessibility for persons with ADRD – capitalizing on relatively well-preserved neurological capacity for music memory and production.^40^ That is, compared to declines in episodic memory and numerous other cognitive functions, select neural systems underlying music cognition remain relatively spared until much later stages of the disease process^41^; in fact, persons with ADRD frequently show the ability to not only enjoy and recognize music, but also perform and engage in music-making activities despite other chronic cognitive impairments.^42,43^ Further, due to the extensive and diverse neural network involved, singing is an especially accessible format for music interventions – notably highlighted by the Alzheimer’s Society’s Singing for the Brain model^44^ and the Silver Song Club project.^45^ These foundational programs help substantiate both the accessibility and enjoyment found in incorporating music into the dementia-care context.

A combination of randomized control trials (RCT), meta-analyses, and qualitative studies have further elucidated the significant benefits of *group* singing specifically for facilitating improvements in cognition, mood, and quality of life in persons with ADRD, while also mitigating deleterious symptoms such as CD and social isolation in caregivers.^23,46-48^ While most group singing interventions focus on either individuals with ADRD or their caregivers singing in their respective peer-groups, dyadic interventions allow for both members of the caregiving dyad to participate concurrently in the same activity. However, such dyadic music interventions are largely under-researched, and there is notable heterogeneity in study-design, measurement outcomes, and methodological approaches among existing studies.^49^ Despite this, compelling research has highlighted how dyadic participation in music interventions can enhance the quality of the relationship between both members in the caregiving dyad.^43,50-52^ Moreover, among the limited empirical investigations, an RCT by Särkämö et al (2013) demonstrated that across a 9-month dyadic music intervention, both singing and listening to music were associated with declines in CD relative to non-intervention peers, with steeper declines noted in the singing group. Caregiver testimonies further highlight the benefits of dyadic music interventions, intimating that such interventions feel like they emphasize making connections with group members and on the joy of singing, as opposed to focusing on the underlying neuropathological process, medication or symptoms of dementia.^53,54^ Dyadic interventions also possess the inherent advantage of offering more flexibility and accessibility to caregivers looking to participate in non-pharmacological interventions, removing the barrier of needing to schedule separate intervention contexts or arrange for temporary care of their loved ones.^55^ All told, dyadic music interventions offer unique value among the non-pharmacological intervention milieu, and continued research is necessary to bolster an understanding of their specific benefits on key psychosocial health outcomes for both caregivers and persons with ADRD.

## The Current Study

Given the relative sparsity of research investigating the impact of dyadic, music-based interventions for dementia caregivers and individuals living with ADRD, this study sought to provide a novel investigation of whether longitudinal participation in a dyadic music intervention facilitates improvements in CD.

The current study leveraged data from the innovative Voices in Motion (ViM) project, an intergenerational community choral intervention incorporating persons with ADRD, their caregivers, and student volunteers. In addition to being an empirically based choral experience, ViM integrates social and leisure components that seek to enhance cognitive function, reduce depression and CD, and increase socialization. Participation consisted of weekly choral sessions within 2 distinct choral seasons – each spanning ∼3.5-month and separated by a summer break. Monthly assessments (consisting of a battery of neuropsychological, cognitive, physiological, and survey metrics) were administered to investigate both between- and within-person change in a variety of health outcomes, including CD.

This longitudinal, intensive repeated measures burst design, with monthly assessments nested within choral seasons, enables a novel evaluation of whether dyadic music interventions facilitate improvements in CD using methods that have not been previously explored in the literature. Specifically, the repeated measures design of ViM allows for a robust estimation of change in CD within choral seasons, both within- and between-persons. Moreover, as the 2 choral seasons were separated by a ∼4-month summer hiatus, in which engagement in the weekly choral sessions and monthly assessments were halted, this presented the further advantage of a naturalistic ABA design. That is, the choir intervention was administered across a participant’s initial choral season (A), removed across an extended summer break (B), and then re-administered for a second choral season (A). This design has not yet been employed in the existing literature on dyadic music interventions but allows for the robust evaluation of whether participation in the choir was directly associated with changes in CD, including whether the cessation of the intervention was associated with the reversion of any potential benefits realized across the first season. These innovations optimally position the ViM project to make novel contributions in understanding dyadic music interventions, helping to evaluate their efficacy in addressing the health and well-being of the rapidly growing community of dementia caregivers.

## Methods

### Participants

The ViM study was approved by the Human Research Ethics Review Board at the University of Victoria (HREB #17-425). Participants in this study consisted of a subsample of 30 caregivers of persons with ADRD (M*age* = 67.7; *SD =* 10.0; *range* = 48-89 years; 80% female, 29 identified as Caucasian) engaged in the ViM choral intervention. Most caregivers lived with their respective care-recipients (77%) and were immediate family members (spouse = 60.00%; adult child = 26.67%; other family = 10.00%, friend = 3.33%).

All ViM participants were recruited through media advertisements (radio, newspaper) and flyers posted at community centers, churches, and doctors’ offices. An initial phone interview assessed eligibility for participation. Participants with ADRD were required to have physician-diagnosed mild or moderate dementia resulting in impairments in activities of daily living, as well as a family caregiver that was equally willing to accompany them and participate in both the choir and research assessments. All participants were provided ample opportunity to pose questions regarding their participation prior to consenting and were informed that could withdraw from the intervention and/or assessments at any time.

The ViM study adopted a rolling sample recruitment strategy where participants were able to join throughout the intervention at the start of each new choral season. This strategy was chosen to maximize accessibility for interested community members and bolster sample size, resulting in 2 distinct cohorts: 1 cohort of participants who were present at the initial outset of the ViM intervention (ie, season 1, assessment 1), and a second cohort that consisted of both returning and new participants following the summer hiatus (ie, season 2, assessment 1). Regardless of when a participant joined, each participant’s baseline assessments equated to their first time in the study, facilitating a meaningful conceptual interpretation of mean performance at study inception (ie, intercept) despite these time-points being different between persons. In relation to the ABA design, this means that all participants contributed data for their initial season (A) of the ViM intervention, but only participants from the first cohort who returned from the summer break (B) provided data for the reintroduction of the intervention across a second choral season (A). The first choral season consisted of 13 caregivers, with 17 new caregivers joining for the second season, yielding a final sample of 30. From the first cohort, 6 caregivers returned to participate in a second choral season. See Table 1 for sample descriptives faceted by cohort.

**Table 1.** Descriptive Statistics for Demographic and Behavioral Data for the First Choral Season

	Cohort 1	Cohort 2
	N *= 13*	N *= 17*
CG baseline age (SD)	67.6 (7.2)	67.8 (11.9)
	*Range = 57-78*	*Range = 48-89*
Person with ADRD baseline age (SD)	78.7 (9.8)	79.8 (7.5)
	*Range = 57-89*	*Range = 70-98*
CG sex at baseline (% female)	69	88
CG relationship (% spouse)	62	59
Person with ADRD MMSE score at baseline (SD)	20.4 (4.9)	20.9 (5.5)
	*Range = 11-26*	*Range = 10-29*
CD at baseline	21.1 (8.4)	15.7 (10.5)
	*Range = 4.25-31.33*	*Range = 0-39.7*
CG social support at baseline (SD)^a^	7.4 (2.7)	7.9 (2.3)
	*Range = 3.5-11.5*	*Range = 3.7-11*
CG depressive symptoms at baseline (SD)^b^	4.2 (2.6)	5.75 (5.1)
	*Range = 0-10*	*Range = 0-18*
Person with ADRD episodic memory score (SD)	3.3 (2.1)	3.1 (3.1)
	*Range = 0-8*	*Range = 0-11*

^a^Self-reported.

^b^PHQ-9 Self-reported.^56^

MMSE = Mini Mental State Examination.^57^

CG = Caregiver; ADRD = Alzheimer’s Disease and Related Dementias; CD = Caregiver Distress.

### Procedure

Participation in the ViM choral seasons consisted of weekly, 1.5-hour professionally conducted choir rehearsals (held at 2 local community churches) followed by 30-minute of socialization. Each choral season lasted approximately 3.5-month and culminated in a final performance for the local community. Songs were selected and directed by the professional conductor (BMus, DipEd, MMus). Song selection deliberately included familiar and popular songs from adolescence and early adult years of the participants to capitalize on the reminiscence bump (ie, songs that have high autobiographical salience), but also included songs in different languages, songs that promoted powerful, positive emotions, and songs that utilized hand and other motor movements.

#### Design

**Figure 1.**
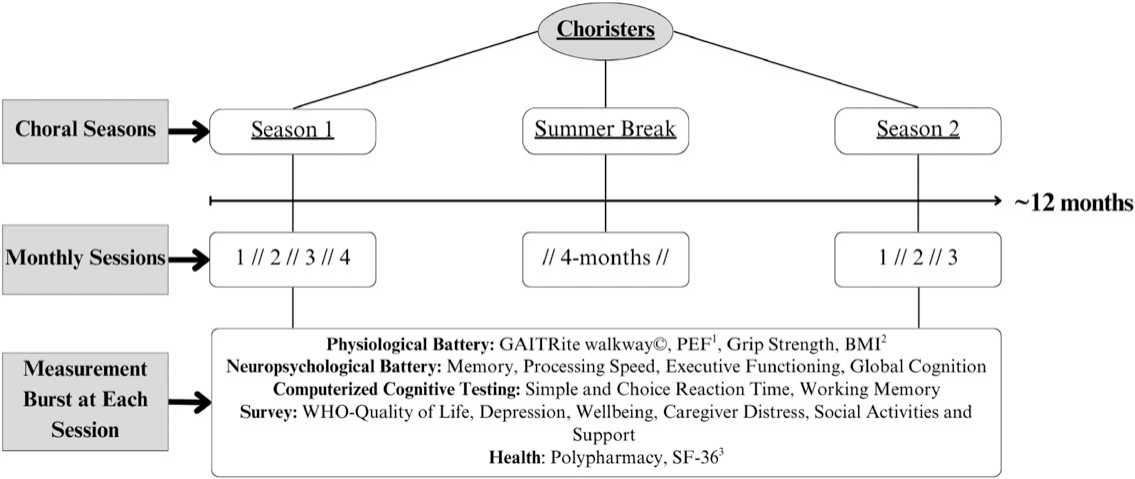
Voices in Motion Longitudinal Measurement Burst Design. *Note.* The Intensive Repeated Measures Burst Design Used by the Voices in Motion Project. Participants Joined Continuously Across Seasons 1 and 2. Each Season Was Comprised of Monthly Physiological, Neuropsychological, Computerized Cognitive, Survey, and Health Evaluations. This Longitudinal Design Allows for a Distinct Parameterization of Between- and Within-Person Effects; Further, The Summer Break Facilitates a Naturalistic ABA Design for Comparing Change Both Across and Within Seasons. ^1^Peak Expiratory Flow; ^2^Body Mass Index; ^3^Short-Form 36^58^

ViM leveraged a longitudinal measurement burst design, where data collection took place at the University of Victoria every 3-4 weeks within each choir season (see Figure 1 for ViM design and testing battery). To facilitate convenience of assessment, decrease the likelihood of study attrition, and mitigate testing effects for persons with ADRD (eg, time of day^59^), participants chose a date and time for assessments - within pre-determined testing windows - that best suited their schedule. This technique also naturally invoked a variable retest interval design, which provides a more accurate evaluation of individual change information.^60^ The entire assessment battery indexed numerous cognitive, physiological, and psychological processes, with the present study focused exclusively on CD. As ViM is an ongoing intervention, the current study used data collected across the first 2 choral seasons, yielding up to 7 assessments per person.

Notably, after the conclusion of the first choral season there was an extended break, which corresponded to an approximate 4-month hiatus over the summer, followed by the beginning of the second choral season; thus, in combination with a measurement burst approach to capture intraindividual change information, this study also used a naturalistic ABA design. This paradigm allows researchers to explore whether systematic reductions in CD are noted within and across 2 separate choral seasons (A), and whether the removal of the ViM intervention (B) was associated with any reversion in CD improvements that may have manifested across the first choral season. These methodological strengths place a novel emphasis on studying within-person change across time, rather than investigating between group differences which has been the focus of previous RCT’s investigating music interventions.

### Measures

#### Caregiver Distress (CD)

The 12-item Short Zarit Caregiver Burden Interview (ZBI^61-63^) was used to measure the level of distress experienced by each caregiver. On a scale ranging from 0 (*never*) to 4 (*nearly always),* caregivers self-reported their responses to 12 individual prompts (eg, ‘Do you feel strained when you are around your care recipient?’, ‘Do you feel that your health has suffered because of your involvement with your care recipient?’, ‘Do you feel that you don’t have as much privacy as you would like because of your care recipient?’, ‘Do you feel that your social life has suffered because you are caring for your care recipient?’). A total summed score was derived ranging from 0 to 48, with higher scores suggesting greater CD. According to the recommended scoring guidelines for the 12-item ZBI,^63^ scores ranging from 0-10 indicate ‘*no to mild burden*’, scores of 10-20 indicate ‘*mild to moderate burden’*, and scores above 20 reflect ‘high burden.’

#### Episodic Memory

Episodic memory (EM) of persons with ADRD was assessed via performance on an immediate free recall task comprising 30 words.^64^ The word list consisted of 6 taxonomic categories (5 words per category). Participants were required to study the words for 2 minutes and were then asked to write down as many words as possible in 5 minutes. The number of correctly recalled words was recorded.

#### Statistical Procedure

Variance decomposition in CD (proxied by the ZBI) was evaluated based on an initial, fully unconditioned model (ie, without predictors). A fully conditioned, longitudinal multilevel model was then fit to estimate the level and rate of change in CD within each choral season. This was accomplished using two-level multilevel piecewise regression models. Specifically, an initial time-in-study (TIS) variable – operationalized as the number of months a person had been in study – was dichotomized into 2 linear regression segments around the summer breakpoint, such that all TIS values were centered at the maximum TIS value for the first choral season (ie, 3.3-month). This facilitated 2 separate slope segments whereby the first choral season TIS values represented a time-*to*-break slope, and the second season TIS values represented a time-*since*-break slope. Given the rolling sample of ViM, all 30 participants informed the initial time-to-break slope – as this corresponds to participant’s change in CD across their initial choral season – but only returning participants who participated in both choral seasons informed the time-since-break slope. Further, new intercept variables were created to indicate the summer break point. Specifically, in combination with the time-to- and time-since-break slopes, these intercepts facilitated a meaningful interpretation of fixed effects, such that the intercept value for the first choral season reflected the average level of CD immediately prior to the summer break, whereas the intercept for the second choral season corresponded to the average level of CD immediately upon returning from the summer break. To control for the potential influence of cohort differences and the known association between care-recipient cognitive impairment and CD^26,65^ participants’ ViM cohort (1 or 2) and their respective care-recipient’s average EM score were each included as covariates.

Equation (1) demonstrates the modeling of average linear change in CD as a function of the time-to-break (TIS1) and time-since-break (TIS2) slopes, moderated by cohort (COHORT; for TIS1 only) and grand-mean centered EM scores (EM; for both TIS1 and TIS2). The intercepts (INT1 and INT2) are interpretable as the level of CD immediately prior to, and after, the inter-season break, respectively, when all predictors are equal to 0. Altogether, CD scores (ZBIij) for a given individual (i) and assessment (j) were modeled as a function of that individual’s time in study (TIS1) and their CD score (INT1) prior to the break, plus their time in study (TIS2) and CD score (INT2) after the break, plus an error term (e). Person-level covariates (Level 2) included ViM cohort as a moderator of both the pre-break slope (δ_01_) and intercept (δ_21_) and EM as a moderator of pre- and post-break intercepts (δ_22_ and δ_31_, respectively) and slopes (δ_02_ and δ_11_, respectively). Random effects were also modeled, whereby the level-1 residual [Var(e_ij_)] reflects within-person assessment-to-assessment variability, and the level-2 residuals [Var(U_2ij_), Var(U_3ij_)] reflect between-person differences in the level of CD immediately prior to and following the summer hiatus, respectively. Notably, level-2 random effects of slope (ie, individual differences in rates of change across each choral season [TIS1, TIS2]) were also investigated, but were dropped from subsequent models as they yielded a singular fit (ie, they were exceedingly small, suggesting no between-person differences in rate of change). Moreover, 3 persons with ADRD did not have EM information, so the final conditioned model was run on a sample of 27.

Level−1 (Assessments within Season):

$${\text{ZBI}}_{\text{ij}}=\beta_{0i}\left(\text{TIS}1\right)+\beta_{1i}\left(\text{TIS}2\right)+\beta_{2i}\left(\mathrm{INT}1\right)+\beta_{3i}\left(\mathrm{INT}2\right)+e_{\text{ij}}$$

$$\text{Level}-2\;\left(\text{Persons}\right):$$

$$\beta_{0i}=\delta_{00}+\delta_{01}\left({\text{COHORT}}_{i}\right)+\delta_{02}\left({\text{EM}}_{i}\right)$$

$$\beta_{1i}=\delta_{10}+\delta_{11}\left({\text{EM}}_{i}\right)$$

$$\beta_{2i}=\delta_{20}+\delta_{21}\left({\text{COHORT}}_{i}\right)+\delta_{22}\left({\text{EM}}_{i}\right)+U_{2\text{ij}}$$

(1) $$\beta_{3i}=\delta_{30}+\delta_{31}\left({\text{EM}}_{i}\right)+U_{3\text{ij}}$$

All analyses were computed using HLM 8 software^66^ with a two-tailed significance threshold of *P* < 0.05 for the investigation of change in CD within choral seasons, and a one-tailed significance threshold of *P* < 0.05 for the inferential comparison of intercepts given our a priori rationale and directional hypothesis regarding whether CD *increases* across the summer break. Notably, the longitudinal intensive repeated-measures burst design provided several within-person trials, increasing statistical power and reliability despite a modest between-subject sample size.^67^ Moreover, restricted maximum likelihood (REML) was employed as the estimator due to its advantageous properties for working with small samples.^60^ Using REML, models also adjusted for individual differences in number of assessments completed such that the final estimated parameters were weighted towards individuals who contributed more observations.^68^

## Results

To characterize patterns of between- and within-person variation in the data, an initial fully unconditioned model was fit to derive the intraclass correlation coefficient (ICC). Of the total variability in CD, 91% reflected between-person variability whereas 9% reflected variance within-persons across months.

**Figure 2.**
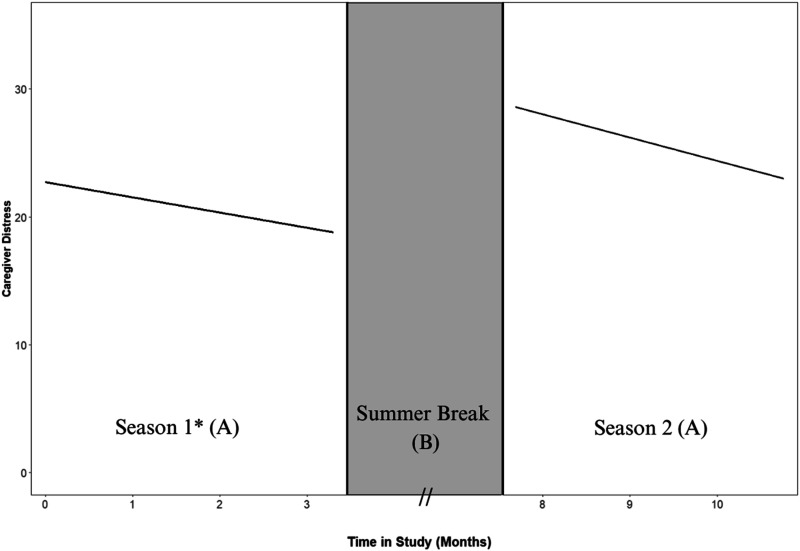
Multilevel Piecewise Regression Models Depicting Change in Caregiver Distress (Proxied by Zarit Burden Interview) Within Choral Seasons. The ABA Design of the Current Study Facilitated an Analysis of Change in Caregiver Distress Across an Initial Choral Season (A), Followed by a ∼4-Month Summer Break (B), and Then Again Across a Second Choral Season (A). *Note.* Linear Spline Slope Estimates: Season 1 = −0.98, *P < 0.05, Season 2 = −1.38, *P* > 0.05. Inferential Comparison of the Intercepts at End of Season 1 (18.8 units) and Beginning of Season 2 (28.5 units) was Also Significant (*P* < 0.05)

To address the first research question regarding change in CD across choral seasons, a multilevel piecewise regression model was fit to evaluate level and change in CD both prior to and following the summer break (see Figure 2). Across the ∼3.5 month first choral season, CD significantly reduced by −0.98 units per monthly session (*P* < .05), with average CD levels declining from 22.73 units at baseline (ie, session 1) to 18.81 units at the session immediately prior to the summer hiatus. In contrast, following the approximately 4-month summer break, the average level of CD at the outset of the second season for returning caregivers was 28.54 units – signifying significantly higher CD (*P* < .05, one-tailed) compared to the end of the first choral season (18.81 units). Notably, care-recipient EM further moderated these average CD levels at the inception of the second choral season, with every unit increase in EM score of care recipients (corresponding to 1 more word recalled on the free recall task above the sample average) associated with a substantial 8.5-unit decrease in CD scores (*P* < 0.05). Finally, upon reintroduction of the ViM intervention in season 2, CD levels began to decline again (−1.38 units per additional monthly retest session), although this trajectory was not significant (*P* > 0.05).

## Discussion

Informal caregivers for persons with ADRD are an integral part of dementia healthcare; however, the surmounting time and effort of caregiving responsibilities, alongside the emotional impact of witnessing a loved-one’s health and functioning decline, can have substantial negative impacts on caregiver health and well-being. Specifically, a myriad of research has showcased the significant levels of distress experienced by caregivers, and the serious implications of CD on the health and well-being of both caregivers and care recipients.^14,17,33,69,70^ Although dyadic music interventions show promise as an accessible and enjoyable intervention option to help reduce CD and facilitate improvements in numerous health faculties, they are largely under-researched among the extant music intervention literature.^49^ The current study leveraged an innovative approach combining the advantages of a measurement burst design – providing up to 7 assessments per participant – and naturalistic ABA structure to provide a novel evaluation of how dyadic singing interventions may impact CD. This design, paired with two-level multilevel piecewise regression models, allowed for the separate parameterization of average rates of change in CD within each choral season, as well as inferential comparisons of CD levels pre- and post-summer break. These 2 innovations permitted the exploration of how CD changed within- and across-choral seasons, and whether the removal of the ViM intervention was associated with a reversion in any CD gains realized across a participant’s first choral season.

Results showcased that CD significantly declined across the first season of the choir, even after controlling for between-person differences in care recipient EM impairment and potential cohort differences. Specifically, CD declined from approximately 23 units at baseline to 19 units at the final assessment of participants’ first choral season. This significant reduction reflects a meaningful improvement in CD across the first choral season, with caregivers transitioning on average from ‘high’ to ‘mild-moderate’ levels of CD. Given the well-documented impact of CD on deleterious cognitive and physical health outcomes for both caregivers and their care-recipients, interventions such as ViM – focused on facilitating such declines in CD – could yield substantial holistic health benefits for the caregiver dyad.

In addition to these notable declines in CD across participant’s first choral season, caregivers who returned for a second season after the summer hiatus exhibited a striking increase in distress levels relative to their scores at the end of the initial season. Specifically, when compared to the average CD level (18.8 units) observed immediately prior to summer hiatus, CD levels at the onset of the second season were nearly 10 units higher (28.5 units). This rebounding of CD scores across the summer gap highlights not only an inferentially significant reversion but represents a magnitude of distress that exceeds even the baseline CD levels. This finding clearly demonstrates the increasing distress associated with caring for someone with an advancing underlying pathology as they develop more complex and intensive needs; indeed, there is a well-documented relationship between the degree of impairment in the care recipient and the level of distress in the caregiver.^26,65^ The current findings support this notion, as individual differences in CD at the outset of the second season were significantly moderated by the EM performance of respective care recipients; specifically, a one-unit decrease in EM performance (corresponding to 1 less word recalled on the free recall task), relative to the sample average, was associated with an 8-unit increase in distress level for caregivers. This finding corroborates evidence on the deleterious impact of declining care recipient health on CD levels, and highlights how interventions aimed at attenuating the behavioral, psychological, and cognitive impairments of persons with ADRD may confer benefits to their caregivers as well.

Furthermore, given the significant declines in CD identified across the first choral season, the rebounding of CD levels across the summer could also be associated with the removal the ViM intervention and its corresponding benefits. Indeed, after the significant increase in CD identified across the summer hiatus, distress levels began to decrease again across the second choral season. Notably, while this effect did not surpass statistical significance, the magnitude of decline was larger than that observed across the first choral season (−1.38 units per session, *P* > 0.05). The failure to meet statistical significance was the likely result of the small sample size of returning participants at the second season; however, the combined findings of (a) significant declines in CD across season 1, (b) significant rebounding of CD across the summer hiatus, and (c) patterns of decline across season 2, provides novel evidence for the benefits of dyadic music interventions in addressing CD.

### The Merits of Dyadic Music-Based Interventions

Previous explorations of music-based interventions, while highlighting numerous health benefits, have largely neglected the exploration of *dyadic* interventions and adopted primarily pre-test post-test designs that precludes more sophisticated longitudinal, within- and between-person insights. To this end, the current study provides several valuable contributions to the literature, highlighting a novel methodological paradigm that explores the longitudinal impact of a dyadic choral intervention on CD across multiple choral seasons. The current results corroborate and expand on extant understandings of the benefits of dyadic music interventions, as well as the impact of dementia severity on CD. Most notably, the significant declines in CD across participant’s initial choral season, combined with the significant reversion in distress levels upon returning from the summer hiatus, suggests not only that dyadic music interventions confer benefits to caregivers, but that removing the support and connection found within such programs facilitates precipitous declines in health and well-being.

The potential mechanisms underlying the positive effects realized by music interventions may be attributable to how they facilitate support, socialization, and modulate negative psychosocial comorbidities in participants.^71^ Music interventions such as ViM naturally promote increased socialization, offer a collaborative and cognitively stimulating activity, and provide a novel and joyful environment relative to other intervention mediums.^43^ Moreover, when participating as a dyad, music interventions have the additional benefits of offering flexibility to participants (eg, not needing to schedule care replacements or juggle multiple individual intervention contexts) and addressing relational strain between members in the caregiving dyad.^43,72^ Indeed, by focusing on promoting social support and engagement, music interventions like ViM have the potential to modulate variables such as CD by addressing and mitigating detrimental comorbidities (such as social isolation and depressive affect) commonly associated with caregiving and dementia.

Alleviating these comorbidities is of continued importance to ViM, which provides prosocial opportunities for persons with ADRD and caregivers to interact and engage, not only with each other, but with an intergenerational network of student and community volunteers as well. This intergenerational element, beyond creating increased opportunity for social connection, also aims to mitigate stigma in the dementia community – a pervasive problem that exacerbates social isolation and further impacts CD.^73^ The regularly scheduled interaction with this diverse social network may partially account for the reductions in CD observed across the first choral season and help explain why – after the summer hiatus where such interactions were removed – CD increased to new highs (ie, 28.5 units). That is, without the consistent socialization opportunities afforded by ViM, and any psychosocial benefits they confer, this may have driven the noticeably large reversion in CD scores across the summer hiatus. Altogether, the current findings highlight the critical influence that the ViM intervention, including its removal, has on CD and provides compelling evidence for the value of dyadic music interventions in addressing the health and well-being of caregiver dyads.

### Limitations

Despite the several strengths and innovations provided by the current investigation, this study is not without limitations. Most critically, the results demonstrated are based on a small sample size, with initial season intercept and slope parameters based on a sample of 27 participants, and season 2 intercept and slope values based upon 6 caregivers. Given that only 6 of the initial thirteen participants in the first ViM cohort 1 returned for a second season, a simple one-way ANOVA was fit to investigate whether these 6 returning participants systematically differed on either their average level of CD or EM impairment of their care recipients relative to those who did not return. This analysis indicated that the 2 groups did not significantly differ on these variables, bolstering confidence that the identified differences in intercepts were not driven by underlying factors related to attrition or fundamental group differences; that is, the large reversion in CD level at the beginning of the second choral season was not due to the returning cohort being fundamentally more distressed, or providing care for more severely cognitively impaired individuals with ADRD, than participants who did not return. Further, to increase the reliability of model estimated parameters, REML was utilized due to it being more robust at dealing with modest sample sizes. Finally, our sample represented a relatively socioculturally homogenous population which may restrict generalizability to the wider dementia population. However, through leveraging the accessibility of an arts-based intervention, our study did include participants that were diverse in age (ranging from 48-89 years), relationship type (spouse = 60.00%; adult child = 26.67%; other family = 10.00%, friend = 3.33%), and included the contribution of male caregivers (20% male), helping provide representation to some underrepresented caregivers in the literature. Moreover, with ViM’s effort to include student and community volunteers, this project adopts an intergenerational, community-focused approach that aims to reduce stigma and broaden the dementia community to include members beyond caregivers and individuals with lived experience.

While replication with a larger and more diverse sample size is necessary to ascertain confidence in the estimated effects, it is important to note that the contributions of the current study aim to stimulate more methodologically innovative investigations of the longitudinal impacts of music interventions on important outcomes such as CD. The combined use of a measurement burst and naturalistic ABA design, with sophisticated multilevel models using piecewise splines, are novel additions to the literature in which multiple assessments per participant allowed for a more robust within-person investigation in CD change.

### Future Directions

The current study showcases the advantages of a dyadic, choral intervention for attenuating CD, which is 1 of a multitude of predictors that are associated with caregiver health and well-being. Future investigations should explore additional biomarkers and behavioral metrics to comprehensively understand the impact of dyadic interventions on caregiver and care recipient health outcomes. Scaffolding on our research methodology, future studies can also facilitate a more nuanced investigation of the complex interplay between factors like CD and care recipient impairment through dyadic analysis, exploring the co-evolution of health processes across time as well as what variables may modulate their influence across intervention participation. It is also important to conduct further mixed methods research to understand factors that relate to participants’ likelihood to participate in music interventions, inclusive of the barriers that impact enrollment or attrition. Addressing these barriers may lead to increased participation, thereby facilitating a wider positive impact on the population.

Finally, the current findings can be mobilized into meaningful health care utilization and knowledge translation. That is, showcasing the clear benefits of dyadic music interventions on reducing CD, and the equally clear detriments of removing such interventions, may stimulate increased support and adoption of comparable intervention strategies from local health authorities and dementia communities. With the growing advocacy of social prescription models for attenuating critical physiological, cognitive and psychosocial challenges faced by caregiving dyads, increasing empirical support for non-pharmacological interventions like ViM will help expedite their recommendation and adoption. The social prescribing of interventions like the ViM project could thereby help attenuate the demands placed on traditional healthcare systems and address the significant mental, physical, and social health challenges faced by dementia caregiving dyads. That is, beyond contributing to methodological innovations in the scientific investigation of dementia choirs, the present research consolidates a growing literature that aims to increase feasible strategies and programs available for dementia caregivers and their care recipients to address salient challenges and comorbidities in their lives. The implementation of effective scientific paradigms is an ongoing challenge within clinical research, but the ViM project aims to address key barriers and facilitate social engagement through the strengths of its design and protocol. Such strengths include the utilization of a rolling sample recruitment approach that allows for flexible onboarding and participation, an inclusive, intergenerational sample that allows for caregivers to participate with their loved ones, including individuals with ADRD, children, grandchildren, or friends (avoiding the need to organize complex scheduling of separate intervention contexts), and an organic, seasonal schedule that aligns with musical conductors, musicians and accompanists professional lives. These components add to the enjoyability and feasibility of social-cognitive interventions like ViM, and, in addition to the empirical support provided by this paper, will help promote its adoption in community contexts.

### Conclusion

The social and financial costs associated with dementia – both on the healthcare system and on informal caregivers –- is substantial and there is a critical need to provide effective intervention opportunities and resources to individuals living with dementia, their caregivers, and health practitioners as part of a social prescription strategy. As a complement to basic pharmacotherapeutic interventions and the ongoing molecular research on dementia, social-lifestyle interventions are a critical element of dementia care. Music-based interventions, such as the ViM Project, address some of the modifiable risk factors that can greatly impact the health and trajectory of both members of a caregiving dyad. Moreover, they offer a social and artistic environment where individuals can collaborate as partners and engage with a different style of intervention. This research provides further support to the efficacy of dyadic, music-based interventions for improving CD across long-term choral seasons through the use of an innovative methodological paradigm and statistical procedure. While further research scaffolding on these initial findings, with larger, more diverse sample sizes and a deeper exploration of the potential mechanisms underlying the choir’s efficacy as an intervention medium are necessary, the current results highlight the clear benefit of music-based treatment programs for both caregivers and their care recipients.

## Supplemental Material

Supplemental Material - Choir Mitigates Distress for Caregivers of Those With Dementia: The Voices in Motion Project Supplemental Material for Choir Mitigates Distress for Caregivers of Those With Dementia: The Voices in Motion Project by Nicholas Tamburri, MSc, Cynthia McDowell, MSc, Carren Dujela, MA, LPN, Mariko Sakamoto, RN, PhD, Denise S. Cloutier, PhD, Jodie R. Gawryluk, PhD, Andre P. Smith, PhD, Debra J. Sheets, PhD, MSN, RN, Robert S. Stawski, PhD, Stuart W. S. MacDonald, PhD in American Journal of Alzheimer’s Disease & Other Dementias®.

## Data Availability

Due to the nature of this research, participants in this study did not agree for their data to be shared publicly, so supporting data is not available. Effort was put towards developing legacy documents to help individuals build similar lifestyle/social-cognitive interventions to ViM within their own communities. Please find these resources publicly available at the ViM YouTube page, located at: https://www.youtube.com/@voicesinmotionchoirs9841. These “Train-the-trainer” resources were designed to help facilitate dementia choirs online during the COVID-19 pandemic. For those interested in further detail, please contact S. W. S. MacDonald (corresponding author and ViM PI).
