# Supplementary material for: Choir Mitigates Distress for Caregivers of Those With Dementia: The Voices in Motion Project
Source: Am J Alzheimers Dis Other Demen. 2025 Nov 6;40:15333175251395437. doi: 10.1177/15333175251395437 (PMC12592667; doi:10.1177/15333175251395437)
Supplement: Supplemental Material - Choir Mitigates Distress for Caregivers of Those With Dementia: The Voices in Motion Project [file sj-pdf-1-aja-10.1177_15333175251395437.pdf]

Supplemental Material:

Table 2. Final Estimates for CD Rate of Change within Choral Seasons 1 and 2, controlling for Episodic Memory Deficits in Care Recipients and Cohort Differences

First Season

	[1]Intercept β_{30}			Slope β_{10}		
	**Coefficient**	**SE**	***p*-value**	**Coefficient**	**SE**	***p*-value**
**TIS**	18.805	2.850	<0.001	-0.981	0.451	0.042
**EM**	-0.822	0.899	0.370	-0.004	0.197	0.982
**Cohort**	-2.895	4.066	0.483	1.241	0.747	0.113

Second Season

	[2]Intercept β_{40}			Slope β_{20}		
	**Coefficient**	**SE**	***p*-value**	**Coefficient**	**SE**	***p*-value**
**TIS**	28.540	5.968	<0.001	-1.381	0.876	0.132
**EM**	-8.526	3.931	0.040	1.197	0.617	0.067

Note:* [1]*Intercept at Season 1 corresponds to average CD level at final assessment of first season.* [2]*Intercept at Season 2 corresponds to average CD level at the first assessment of participants' second choral season. TIS = Time in Study. EM = Episodic Memory, Grand-Mean Centered. Cohort is coded 0 (First ViM cohort) and 1 (Second ViM cohort).
